# Jejunal-Ileal Bypass and its Complications: Case Report and Review of the Literature

**DOI:** 10.7759/cureus.9276

**Published:** 2020-07-19

**Authors:** Arsalan Khan, Anwaruddin Syed, Daniel Martin

**Affiliations:** 1 Clinical Nutrition, OSF HealthCare, Peoria, USA; 2 Internal Medicine, OSF Multispeciality Group, Peoria, USA; 3 Internal Medicine, College of Medicine, University of Illinois, Peoria, USA; 4 Gastroenterology and Hepatology, OSF HealthCare, Peoria, USA

**Keywords:** bariatric surgery, jejunal ileal bypass, gastroenterology, clinical nutrition

## Abstract

Herein, we describe an interesting case of a patient who underwent a jejunal-ileal bypass (JIB). She presented to the hospital with gastrointestinal bleeding after which her hospital course was complicated by electrolyte abnormalities. She was a 76-year-old Caucasian female with a past medical history of hypertension, type II diabetes, atrial fibrillation on warfarin, chronic obstructive pulmonary disease (COPD) treated with 3 liters of oxygen at home, obstructive sleep apnea, and morbid obesity, with history of an unknown type of bariatric procedure done in the 1970s. Her upper endoscopy showed a normal esophagus, stomach, and duodenum despite her history of bariatric surgery. Her colonoscopy revealed stenotic terminal ileum and an incidental colonic anastomosis at 35 cm from the anal verge with spot tattoo ink of unknown significance. Also noted were moderate internal hemorrhoids and large external hemorrhoids which were likely the source of her bleeding. Post endoscopy she had marked derangement in electrolytes, specifically hypocalcemia, hypomagnesemia, and hypo-phosphatemia.

JIB was first popularized in the 1960s for the treatment of obesity. There are two variations of the procedure, colloquially known as the Scott bypass and the Payne bypass. Our patient underwent the Scott JIB. The relatively longer intestinal tract combined with her ileal stenosis may explain her 50 years of relatively stable adaptation. It is imperative that treating physicians have a keen understanding of anatomy and physiology to adequately care for the long-term needs of these patients.

## Introduction

Obesity is a growing epidemic in the United States. The estimated prevalence of obesity currently stands at 39% of the adult population [1]. This growing epidemic has led to an increased interest in surgical weight loss procedures. Modern bariatric surgeries evolved from antiquated procedures such as the jejunal-ileal bypass (JIB). Although the older procedures are no longer performed, there are still living patients who may have undergone them in the past. One of the earliest bariatric procedures was the JIB of which there were two variants. The bariatric procedure was first popularized in the 1970s and was quickly abandoned due to the high rate of life-threatening complications [2]. We describe an interesting case of a patient who underwent a JIB in the 1970s who presented to the hospital with gastrointestinal bleeding requiring endoscopic evaluation. Her post-endoscopy course was complicated by severe electrolyte abnormalities worsened by malabsorption due to altered anatomy.

## Case presentation

A 76-year-old Caucasian female patient with a past medical history of hypertension, type II diabetes, atrial fibrillation on warfarin, COPD on 3 liters of oxygen at home, obstructive sleep apnea, and morbid obesity, with history of an unknown type of bariatric procedure performed in the 1970s presented to the hospital with complaints of bright-red blood mixed with painless stool for the past six days. On presentation, she denied any nausea, vomiting, abdominal pain, or hematemesis. She did not complain of any fever or chills, chest pain, shortness of breath, cough, hematuria, changes in her vision, dysgeusia, anosmia, or any focal weakness. Her past surgical history included cholecystectomy and appendectomy. She lives at home with her son who assists her with activities of daily living.

On presentation, she had stable vital signs with a weight of 231 lbs, height of 5’0’’, and a body mass index of 45. Generally, she was ill-appearing though not in any acute distress. Oral, pulmonary, and cardiac exams were unremarkable. The abdomen was soft, non-tender; bowel sounds were normal. The digital rectal exam showed large nonbleeding external hemorrhoids. Her extremities had trace pitting edema. The neurological exam was unremarkable with no horizontal or vertical nystagmus. 

Her upper endoscopy showed a normal esophagus, stomach, and duodenum despite her history of bariatric surgery. Her colonoscopy revealed stenotic terminal ileum and a colonic anastomosis at 35 cm from the anal verge with spot tattoo ink of unknown significance. Also noted were moderate internal hemorrhoids and large external hemorrhoids which were likely the source of her bleeding. 

Post endoscopy she had marked derangement in electrolytes, specifically hypocalcemia, hypomagnesemia, and hypo-phosphatemia which was suspected to have occurred due to colonoscopy prep [Table 1]. This complaint along with her uncertain history of bariatric surgery prompted consultation to the nutrition support services. 

**Table 1:** Data from laboratory tests

Variable	Observed value	Reference range
Complete blood count		
White blood cells	21,390/mcL	(4,000-12,000/mcL)
Hemoglobin	11.2 g/dL	(12-16 g/dL)
Platelets	339,000/mcL	(140,000-440,000/mcL)
Coagulation		
International normalized ratio	5.3	(0.9-1.2)
Erythrocyte sedimentation rate	normal	
Stool test		
Fecal calprotectin	normal	
Complete metabolic panel		
Sodium	142 mmol/L	(136-145 mmol/L)
Potassium	2.6 mmol/L	(3.5-5.1 mmol/L)
Creatinine	2.02 mg/dL	(0.6-1.4 mg/dL)
Albumin	3.8 g/dL	(3.5-5 g/dL)
Calcium	5.2 mg/dL	(9.1-10.5 mg/dL)
Total bilirubin	1.3 mg/dL	(0.2-1.2 mg/dL)
Aspartate aminotransferase	42 U/L	(5-34 U/L)
Alanine aminotransferase	22 U/L	(0-55 U/L)
Alkaline phosphatase	62 U/L	(40-150 U/L)
Lactic acid	3.8 mmol/L	(0.7-2.0 mmol/L)
Magnesium	0.7 mg/dL	(1.6-2.6 mg/dL)
Vitamin and mineral levels		
Zinc	0.49 mcg/mL	(0.66-1.10 mcg/mL)
Copper	0.67 mcg/mL	(0.75-1.45 mcg/mL)
Vitamin A	12.2 mcg/dL	(32.5-78 mcg/dL)
Vitamin E	3.1 mg/L	(5.5-17 mg/L)
25-hydroxy vitamin D	4 ng/mL	(>30 ng/mL)

**Figure 1:**
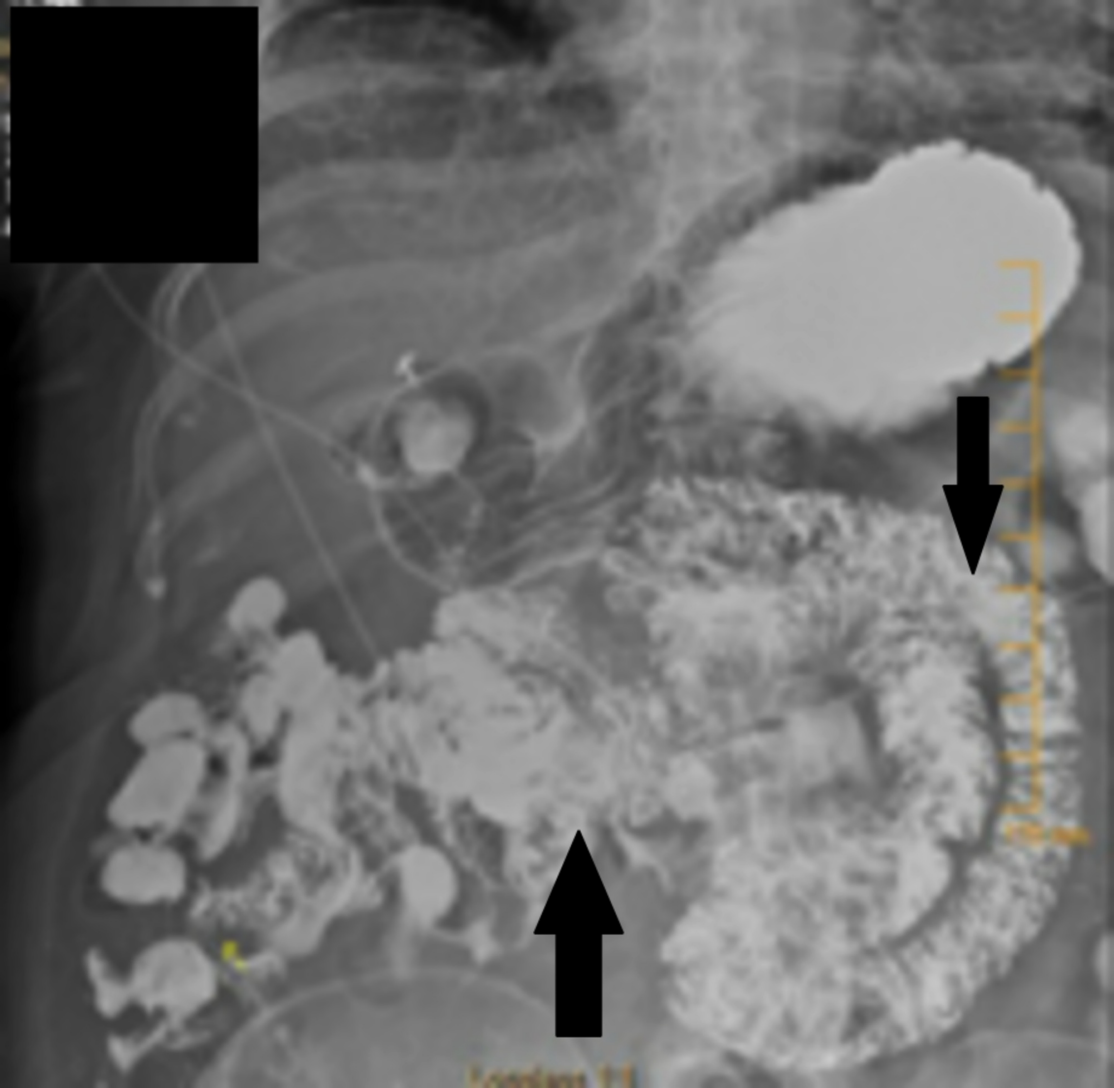
X-ray barium small bowel follow-through with arrows showing opacified and non-opacified small bowel loops

**Figure 2:**
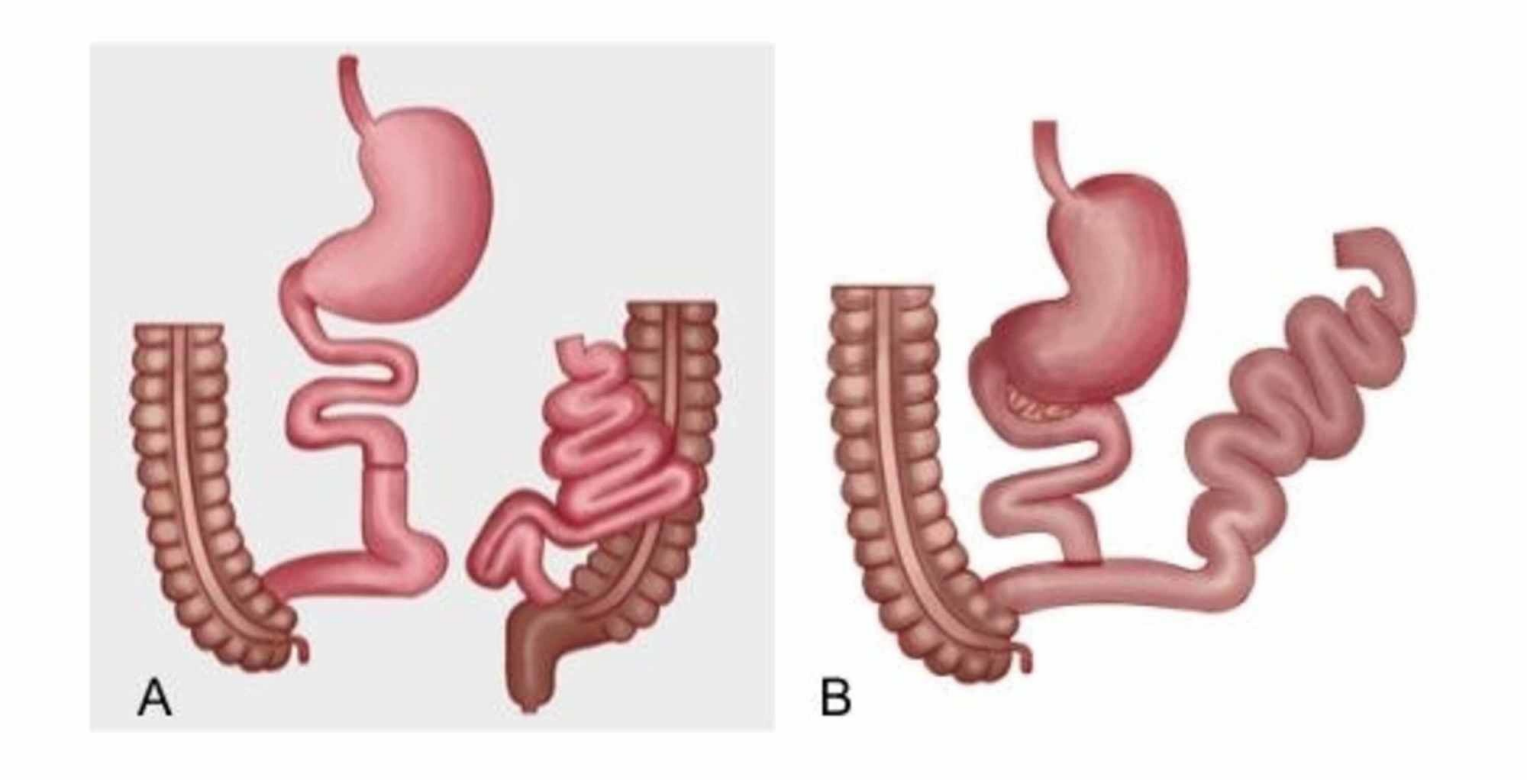
(A) Payne and DeWind Jejunoileal bypass, (B) Scott Jejunoileal bypass

A thorough review of the patient’s history and hospital course was conducted. She insisted that she had a 'stomach bypass' done in the past, despite a normal-appearing stomach on upper endoscopy. At this point, she was suspected of having a jejunal-ileal bypass. A barium follow-through showed non-opacification of a portion of small bowel loops confirming her altered anatomy (Figure 1). Retrospectively, the colonic anastomosis seen on lower endoscopy was likely her excluded small bowel (Figure 2B). Her JIB resulted in malabsorption leading to marked electrolytes derangements exacerbated by the bowel prep. She was managed conservatively with electrolyte monitoring and replacement. Her electrolytes stabilized over the next several days. 

Imaging findings

The patient's esophagogastroduodenoscopy findings showed a normal esophagus, stomach, and duodenum with no evidence of any ulcers or bleeding. The colonoscopy identified a stenotic-appearing terminal ileum that could not be intubated; biopsies were obtained via passing forceps through the IC valve. Other findings include colonic anastomosis at 35 cm from the anal verge with spot tattoo ink, moderate internal hemorrhoids with high-risk stigmata as well as large external hemorrhoids. The video capsule endoscopy results were unremarkable. Pathology from the terminal ileum showed mild villous blunting but no active inflammation or malignancy.

## Discussion

JIB was first popularized in the 1970s for the treatment of obesity. There were two variations of the procedure, colloquially known as the Scott bypass and the Payne bypass. Paye and DeWind described an end-to-side jejunoileostomy that preserved a small segment of the jejunum and a small length of terminal ileum (Figure 1A). The Scott bypass was a variation of the previously described procedure which entails an end to end anastomosis between the jejunum and ileum with the excluded small bowel anastomosed to the colon (Figure 1B) [3-4]. Our patient underwent the Scott JIB, the relatively longer intestinal tract, and delayed transit due to ileal stenosis may explain her fifty years of relatively stable adaptation. Despite this however patients who have undergone JIB remain at risk of multiple medical complications. Here we will review some of the more common issues in the treatment of these patients. 

Diarrhea and electrolyte imbalance

JIB, like most other bariatric procedures, can result in significant diarrhea and electrolyte abnormalities. Diarrhea in such patients is multifactorial but is usually related to malabsorption. The iatrogenically shortened bowel does not allow for appropriate absorption of fat and other nutrients resulting in steatorrhea. In addition, loss of gastrointestinal endocrine hormones, because of altered anatomy, contributes to the malabsorption by loss of ‘intestinal braking’ leading to rapid transit thereby worsening diarrhea [2,4-5]. Electrolyte loss post-bariatric surgery is directly related to the degree of malabsorption and diarrhea. The proximal small bowel receives up to 8L of fluid and electrolytes daily of which most is reabsorbed distally. Cases of iatrogenic shortened bowel, such as bariatric procedures, can, therefore, result in life-threatening losses of sodium, potassium, magnesium, and phosphorous [2,5]. In these patients caution and close clinical observation is warranted when prepping for a colonoscopy.

Cholelithiasis

Cholelithiasis following bariatric procedures is a well-known complication. The exact etiology of stone formation is not well defined however may be related to the release of cholesterol from tissue following rapid weight loss. This excess cholesterol is excreted via bile resulting in increased lithogenicity of the bile [6]. The actual etiology however is likely more complex as some studies have found no relationship between the degree of weight loss and bile stone formation [7]. Altered anatomy in this patient population makes endoscopic-retrograde-cholangiopancreatography (ERCP) a challenge as most endoscopes will not have sufficient length to reach the ampulla. Though time-consuming, laparoscopic-assisted ERCP remains a viable option. 

Renal stones

Renal stones are a common occurrence following bariatric procedures as the altered anatomy results in enteric hyperoxaluria. Undigested fat in the colon binds calcium thereby displacing oxalate from calcium. Oxalate is then absorbed in the colon and excreted via urine. This ultimately results in the formation of oxalate kidney stones. In JIB the incidence of oxalate kidney stones approaches 30% over 15 years [8-9]. Prevention of oxalate kidney stones in bariatric surgery patients consists of a low-fat diet, to reduce the degree of malabsorption, and calcium supplementation to bind oxalate and prevent its absorption in the colon. 

Metabolic bone disease 

Metabolic bone loss occurs to some degree after nearly every known bariatric surgery procedure. The degree of loss of bone mineral density seems to correlate with the degree of weight loss following surgery. This loss of bone density is multifactorial resulting from inadequate vitamins, minerals, protein absorption, as well as hormone alterations leading to bone resorption [10]. In jejunal-ileal bypass patients specifically, osteomalacia approaches nearly 50-60% of patients [11]. Post-bariatric procedure patients should undergo yearly calcium, OH-Vitamin D, PTH, and DEXA scans [12-13]. Patients should have adequate supplementation if any deficiencies are noted. If a patient develops osteoporosis medical therapy with antiresorptive, should be considered. Bisphosphonates are the mainstay of antiresorptive therapy in this patient population and should only be initiated after ensuring adequate levels of calcium, PTH, and 25-OH vitamin-D due to the high risk for hypocalcemia [14].

Liver failure

Liver damage following bariatric surgery is relatively uncommon since the abandonment of JIB. In JIB incidence of liver injury approached up to 10% of patients [15-16]. Liver damage in such cases is multifactorial stemming from pre-existing liver disease, altered metabolism of nutrients, and small intestinal bacterial overgrowth (SIBO). 

Nonalcoholic fatty liver disease (NAFLD) is the most common form of liver disease in obese patients undergoing bariatric surgery. Patients with NAFLD may have normal preoperative liver function testing despite having underlying pathology. Following bariatric surgery, there is rapid weight loss leading to increased free fatty acid influx into the liver making it vulnerable to oxidative stress. This stress causes lipid peroxidation, hepatocyte degeneration, and release of pro-inflammatory cytokines. In addition, hypoproteinemia further decreases the hepatic antioxidative capacity and worsening of liver disease [17-19]. 

Lastly, SIBO may occur following bariatric procedures, especially those that result in excluded bowel and may in fact contribute to liver injury. The dysbiosis caused by the altered anatomy leads to impaired barrier function which may result in liver injury through increased proinflammatory cytokines. In addition, the overgrowth of bacteria results in deconjugation of bile salts which can lead to worsening vitamin and mineral absorption, specifically fat-soluble vitamins [18, 11].

Treatment of liver impairment following jejunal-ileal bypass consists primarily of the prompt reversal of the procedure [9].

Vitamin/mineral levels

Vitamin and mineral levels are routinely monitored in all patients following bariatric surgery. Deficiencies are more common in malabsorptive procedures however can occur following restrictive procedures as well. Table 2 contains a review of common vitamin and mineral deficiencies following bariatric procedures and their treatment [19-20].

**Table 2:** Common vitamin and mineral deficiencies following bariatric procedures IU: international units; PO: per oral

	Definition	Symptoms of Deficiency	Treatment
Vitamin A	Serum retinol <20-80 ug/dL	Night blindness, Bitot spots, hyperkeratinization of skin	10,000-25,000 IU PO daily, higher in situations of corneal damage
Vitamin D	Serum 25-OH <20 ng/mL	Osteomalacia, muscle aches, low back pains	50.000 IU Q-weekly 6-8 weeks
Vitamin E	Serum Alpha-tocopherol <5 μg/mL	Hyporeflexia, neurologic damage, muscle weakness,	100–400 IU/d
Vitamin K	Elevated PT	Bleeding, bruising	1–2 mg/d orally
Zinc	Plasma zinc 60–130 ug/dL	Dysgeusia, rash/acne, diarrhea, hypogonadism	Optimal dosing unclear, usually 50 g (elemental) PO daily
Copper	Serum copper 11.8 to 22.8 mmol/L	Anemia, neutropenia, pancytopenia, hypopigmentation	3–8 mg/d oral copper gluconate
B12	Serum B12 200–1000 pg/mL	Glossitis, pernicious anemia, tinnitus	1000mg/d

## Conclusions

The jejunal-ileal bypass is a unique bariatric procedure that many physicians have relegated to a bygone era. Given the severe complications attributable to this procedure, many of the patients that underwent it have either died of its complications or undergone reversal. Anecdotally many of the patients who underwent JIB had subsequent reversals due to complications associated with this surgery. These patients are precariously balanced such that any stress can lead to significant and potentially life-threatening alterations in electrolytes or vitamin/mineral status. It behooves the clinician to identify such patients and understand their physiology and anatomy. In our case, the patient was able to recover and maintain her electrolyte balance. Her vitamin deficiencies were corrected with oral replacement. Given her advanced age, obesity-related comorbidities, and a 50-year history of successful bowel adaptation, the decision was made not to proceed with a reversal. To date, she continues to follow up with the nutrition support service for routine monitoring.
